# Association Among Self‐Compassion, Resilience, Positive Mental Health and Risk of Gaming Disorder in 18–30 Year Old Population in China and Thailand: A Cross‐Regional Study

**DOI:** 10.1002/nop2.70353

**Published:** 2026-02-08

**Authors:** Anson Chui Yan Tang, Regina Lai‐Tong Lee, Paul Hong Lee, Winnie Lai Sheung Cheng, Shun Chan, Yufang. Guo, Yan Wang, Qing Wang, Pimpimon Wongchaiya, Lorna Kwai Ping Suen

**Affiliations:** ^1^ School of Nursing Tung Wah College Hong Kong China; ^2^ School of Nursing and Midwifery, College of Health, Medicine and Wellbeing University of Newcastle New South Wales Australia; ^3^ Southampton Clinical Trials Unit University of Southampton Southampton UK; ^4^ S.K. Yee School of Health Sciences Saint Francis University Hong Kong China; ^5^ School of Nursing and Rehabilitation Shandong University Shandong China; ^6^ Macao Polytechnic University Macau China; ^7^ School of Nursing Lanzhou University Lanzhou China; ^8^ Faculty of Nursing Chiang Rai Rajabhat University Chiang Rai Thailand

**Keywords:** positive mental health, resilience, risk of GD, self‐compassion, young adults

## Abstract

**Aim:**

The existing literature typically reports an association between Gaming Disorder (GD) and psychopathological outcomes. However, evidence linking positive psychological attributes with the risk of GD is rarely explored. The present study aimed to investigate the associations between resilience, positive mental health and self‐compassion and the risk of GD in young adults.

**Design:**

The study was cross‐sectional, collecting data online in four cities across China and one city in Thailand.

**Methods:**

Potential participants were recruited through social media, gaming platforms and tertiary institutions or universities. The online survey sought information on demography, gaming behaviours, risk of GD, level of self‐compassion, resilience and positive mental health. A General Linear Model was used to compute the associations between the study variables. A *p* value less than 0.05 is regarded as statistically significant.

**Results:**

A total of 1750 young adults were recruited. The results showed that self‐compassion and positive mental health were negatively associated with GD but that was not the case for resilience. Young adult gamers with lower levels of self‐compassion and moderate mental health had a greater risk of gaming addiction as compared to those with a higher level of self‐compassion and flourishing mental health. This is the first study to report the association between GD and three positive psychological factors. The preliminary findings suggest that self‐compassion and positive mental health could be protective mechanisms against GD. Primary healthcare professionals could include self‐compassion and positive mental health in health screening for the early identification of gamers at risk of GD before they manifest psychopathological symptoms. Future studies of GD should explore the effect of positive psychological factors in managing GD in addition to the psychopathological approach.

**Patient or Public Contribution:**

No patient or public contribution.

## 1 Introduction

Digital or video gaming is a ubiquitous leisure activity in the new digital era. Excessive or uncontrollable gaming could be pathological. Gaming Disorder (GD), which is the official name for all kinds of addictive gaming, has been explicitly defined in the *Diagnostic and Statistical Manual of Mental Disorders V* (DSM‐V) and the *11th Revision of the International Classification of Diseases* (ICD‐11) (American Psychiatric Association 2013; World Health Organization 2020). To date, systematic reviews have shown that the prevalence rate of GD in Thailand and China is much higher than the global average of 2% (Paulus et al. 2018). The pooled prevalence rates in Thailand and China were 5.7% and 14%, respectively (Chia et al. 2020; Liao et al. 2022). People aged 18–30 had a pooled prevalence rate of 10.4%, which was significantly higher than the level of 8.8% observed in the adolescent population (Gao et al. 2022). GD has been found to be associated with many negative psychosocial consequences such as depression, anxiety, bullying, interpersonal problems and suicidal behaviour (Gao et al. 2022; Ji et al. 2022). The search for effective measures to identify, prevent and manage GD must therefore be prioritised.

Research has found that gamers with psychopathology, greater vulnerability to stress and dysfunctional coping styles are susceptible to addictive gaming because they tend to cope with negative emotion provoked by daily stressors by immersing themselves in the game world (Brand et al. 2016). These gamers exhibit poor emotion regulation when dealing with the challenges of everyday life (Sulaksono et al. 2020). Better emotion regulation can be achieved when a person enriches their psychological resources to promote psychological well‐being (Morrish et al. 2018). Self‐compassion, resilience and positive mental health have been found to be significantly associated with emotion regulation (Finlay‐Jones et al. 2015; Scoglio et al. 2018; Kraiss et al. 2020; Polizzi and Lynn 2021). Self‐compassion negatively predicted difficulties in emotion regulation and stress symptoms (Finlay‐Jones et al. 2015). Scoglio et al. (2018) found that self‐compassion was negatively related to emotion dysregulation. A meta‐analysis revealed that impaired emotion regulation showed a negative moderate correlation with mental well‐being in patients with mental disorders (Kraiss et al. 2020). A systematic review showed that emotion regulation was positively associated with resilience (Polizzi and Lynn 2021). However, the associations between these three positive psychological attributes and GD are poorly understood due to limited available evidence.

### 1.1 Self‐Compassion

Self‐compassion is a Buddhist concept which involves avoiding narrow, rigid self‐judgement and applying discriminating wisdom to personal wrongdoing. It entails being kind and caring to oneself in the face of hardship, being open to suffering, using a non‐judgemental attitude towards one's own inadequacies and failures and recognising all of these as part of the common human experience (Neff 2003; Neff et al. 2007). It is rarely investigated in studies related to GD. Self‐compassion has three key components: (1) self‐kindness, meaning that an individual gently and patiently encourages his/her ego to change behaviours, rather than employing harsh criticism and judgement (2) common humanity, which means seeing one's experiences as part of the larger human experience rather than as separate and isolating; and (3) mindfulness, which involves holding painful thoughts and feelings in balanced awareness rather than over‐identifying with them (Neff 2003). Self‐compassion is regarded as an effective strategy for emotion regulation because shifting focus from personal failures to self‐kindness/understanding and shared humanity can circumvent negative affect and create more positive emotions (Neff and Seppala 2016). It also enhances a person's resilience to adversities (Neff and Seppala 2016).

Studies on substance abuse and Internet addiction support the positive effect of self‐compassion in reducing addiction risk. Phelps et al. (2018) found that high risk of substance abuse was strongly negatively correlated with all components of self‐compassion. Another drug abuse study found that self‐compassion was negatively related to drug cravings in clinical samples (Shahin et al. 2021). Wisener and Khoury (2020) showed that self‐compassion had an indirect effect on alcohol‐ and marijuana‐related problems by reducing coping‐motivated use of drink and drugs as an attempt to deal with anxiety. Iskender and Akin (2011) investigated the relationship between self‐compassion and Internet addiction in university studies. They found that Internet addiction was negatively predicted by self‐kindness and mindfulness, which are two of the three components of self‐compassion described above. By contrast, all three antitheses of self‐compassion—self‐judgement, self‐isolation and over‐identification—were positive predictors of Internet addiction.

As can be seen, the current literature suggests a potential linkage between self‐compassion and some addictive disorders, making an association between self‐compassion and GD plausible.

### 1.2 Resilience

Resilience is the ability of an individual to bounce back from or cope successfully with stress or difficult situations (Masten 2014). A systematic review and meta‐analysis reported a weak correlation between resilience and problematic Internet use (Hidalgo‐Fuentes et al. 2023). People with a high risk of smartphone addiction have been found to have lower resilience compared to those with low or no risk (Kim et al. 2014; Wisniewski et al. 2015). As regards GD studies, some recent research work investigating resilience in Internet Gaming Disorder (IGD) suggested that resilience may be negatively associated with the severity of IGD. Shin et al. (2019) and Yen et al. (2019) conducted studies in Taiwan and Korea to investigate IGD and its relationship to resilience and other psychological correlates. Both studies reported that resilience was negatively associated with IGD and its symptoms. However, the small sample sizes (*n* = 151–174), skewed gender samples and failure to measure GD by the official diagnostic criteria compromised the validity of the findings. A study with a larger sample size, including both male and female participants and measuring GD by the official diagnostic criteria is needed in order to verify those findings.

### 1.3 Positive Mental Health

Previous research has frequently reported a positive association between GD and negative mental health outcomes but has rarely examined the effect of the positive component of mental health on GD. The World Health Organization has defined mental health as ‘a state of well‐being in which the individual realizes his or her own abilities to cope with the normal stress of life, work productively and fruitfully and be able to make a contribution to his or her community’ (World Health Organization 2021). In accordance with this definition, mental health is currently perceived as a continuum between two extreme mental states: positive mental health and mental illness (Keyes et al. 2010). Keyes (2002) defined positive mental health as ‘a syndrome of symptoms of positive feelings and positive functioning in life’ (p. 1). Positive mental health is important for an individual to be able to maintain a ‘flourishing’ life, which is defined as having a high level of positive emotion and as functioning well psychologically and socially and which may protect a person against mental health problems (Chida and Steptoe 2008; Huppert 2009). By contrast, those with an incomplete positive mental health state are regarded as having a ‘languishing’ life, where the individual neither feels good nor functions well in life and is at risk of mental illness (Keyes et al. 2010). Positive mental health was operationalised by Keyes (2002, 2007) in terms of emotional, psychological and social well‐being, including individuals' perceptions and evaluations of their own lives and their abilities to function well in life. Psychological well‐being emphasises the development of individual potentialities and the completion of evolutionary tasks that occur during development, such as self‐acceptance and forging positive relations with others. Emotional well‐being refers to emotional responses, life satisfaction domains and global judgements on life satisfaction. It includes three main components: life satisfaction, positive affect and negative affect. Social well‐being is the ability to demonstrate a positive attitude and acceptance towards individual differences, to feel confident that society and its members can evolve positively and to perceive one's activities as useful and relevant to society (Keyes 2002, 2007).

A Canadian study showed that positive mental health was moderately correlated with mental disorders and substance disorders (Gilmour 2014). It found that a significantly higher proportion of substance users were classified as having languishing mental health than as having moderate or flourishing mental health states (Gilmour 2014). In social media addiction studies, positive mental health was negatively associated with addictive use of social media (Brailovskaia et al. 2019; Brailovskaia and Margraf 2022). Social well‐being and psychological well‐being were found to be negatively correlated with Internet‐related addiction (Sari et al. 2019). These findings suggest that positive mental health may also be a potential associated factor of GD.

### 1.4 The Present Study

The present study sought to investigate the associations between self‐compassion, resilience, positive mental health and the risk of GD in young adults aged 18–30. The hypotheses are:

Hypothesis 1 *Self‐compassion is negatively associated with the risk of GD among adults aged 18–30*.

Hypothesis 2 *Resilience is negatively associated with the risk of Gaming Disorder among adults aged 18–30*.

Hypothesis 3 *Positive mental health is negatively associated with the risk of Gaming Disorder among adults aged 18–30*.

### 1.5 Significance of the Study

The findings of this study offer empirical evidence to substantiate associations between positive psychological attributes related to emotion regulation and the risk of GD. The findings provide insight, which can be of use in mental health research and practice, about the potential protective effects of self‐compassion, resilience and positive mental health against GD. Clues are provided for future studies that investigate GD from a positive psychological perspective. At a practice level, the established associations may facilitate community nurses and other social and healthcare professionals in identifying gamers at risk of addictive gaming based on low self‐compassion, low resilience and a poor positive mental health state as compared with normal gamers.

## 2 Methods

### 2.1 Study Design and Setting

Data for this cross‐sectional survey was collected in China from December 2021 to April 2022 and in Thailand from August 2022 to February 2023. The data was collected in four cities in China (Hong Kong, Lanzhou, Shandong and Macau) and in the city of Chiang Rai in Thailand.

### 2.2 Participants and Procedure

Potential participants were recruited by convenience sampling through tertiary institutions and universities and social media platforms such as Facebook, Instagram, gaming forums and discussion boards in the focal cities. The inclusion criteria were that individuals aged 18–30 and had experience playing digital/video games for at least 12 months. Those with diagnosed psychological or mental disorders, such as anxiety disorder, depression and regular use of psychotic drugs, were excluded from the study. The required sample size was 645 to achieve a study power of 0.8, α level of 0.05, small effect size (*f* ^2^) of 0.02 and five independent variables (Cohen 1992). GPower ver. 3.1.97 was used to calculate the sample size.

Data was collected using an online survey platform. The first page of the online survey stated the purpose, procedure and potential benefits of the study. The participants were screened for eligibility after giving consent online; those who failed to meet any of the selection criteria were unable to access the survey. The survey contained six sections: demographic information, gaming behaviours, validated measurement scales for the risk of GD, positive mental health, resilience and self‐compassion. The survey took 20–25 min to complete.

### 2.3 Measures

#### 2.3.1 Risk of Gaming Disorder

The main outcome of interest in this study is the risk of GD. It was measured by the Chinese and Thai versions of the 9‐item Internet GD Scale (IGDS) (Lei et al. 2020; Boonyaprasert and Kiatrungrit 2021), which were translated from the original English version developed by Lemmens et al. (2015). The nine items were derived from the nine diagnostic criteria of Internet Gaming Disorder (IGD) in *DSM‐V* (American Psychiatric Association, DSM‐5 Task Force 2013). The items include such questions as, ‘During the last year, have there been hour‐long periods when all you can think of is the next time you can play a game?’, ‘During the last year, have you kept the time you spent on game playing secret from others?’ and ‘During the last year, have you had serious problems with family, friends or a partner because of gaming?’. As the original scale is used to measure only IGD, the wording of the items was modified where necessary to include both digital and video games. The participants were asked to indicate whether they had experienced any of the symptoms described by the nine items over the past 12 months, using a dichotomous scale (1 = yes, 0 = no). The total GD score was the sum of the ratings for all nine items, ranging from 0 to 9. A higher score indicates a higher risk of GD. The risk of GD was also classified into three levels according to Lemmens et al. (2015): normal gamer (score 0–1), at‐risk gamer (score 2–5) and disordered gamer (score 6–9). The reliability and validity of the Chinese version were tested in two population‐based samples of Chinese adolescents and adults (Lei et al. 2020). The results demonstrated good internal consistency, with Cronbach's α of 0.89 and test–retest reliability with r of 0.84. Validity was also indicated by significant correlations with measurements of Internet addiction, aggression, impulsivity, craving for gaming and time spent playing games (Lei et al. 2020). The reliability of the Thai version was found to be acceptable, with Cronbach's α of 0.69. Validity was tested by comparing the total GD score with the DSM‐V diagnostic criteria for Internet GD and ICD‐11 diagnostic criteria for GD, which demonstrated good sensitivity and specificity (Boonyaprasert and Kiatrungrit 2021).

#### 2.3.2 Gaming Behaviour

Data on daily gaming time and years of gaming was collected using an ordinal scale. Daily gaming time was operationalised as 1–2 h, 3–6 h, 7 h or above. Years of gaming was measured by four categories: 1–3 years, 4–6 years, 7–9 years and 10 years or above.

#### 2.3.3 Positive Mental Health

Positive mental health was measured using Chinese and Thai versions of the Mental Health Continuum Short Form (MHC‐SF) (Chinese version: Guo et al. (2015); Thai version: our translation based on the English version). The MHC‐SF measures the three components of positive mental health using 14 items: six items for psychological well‐being (e.g., ‘You liked most parts of your personality.’, ‘You had warm and trusting relationships with others.’); five items for social well‐being (e.g., ‘You had something important to contribute to society.’, ‘People are basically good.’); and three items for emotional well‐being (e.g., ‘happy,’ ‘interested in life’). Based on their feelings over the past month, the participants rated these items on a 6‐point Likert scale, from ‘Never’ (0) to ‘Every day’ (5). The total positive mental health score ranged from 0 to 70 and was categorised into three states of positive mental health, that is, flourishing, moderately mentally healthy and languishing, according to Lamers et al. (2011). The Chinese version was psychometrically examined by Guo et al. (2015). Cronbach's α was above 0.8 for both the total and subscales, indicating good reliability. Good convergent and discriminant validity were also demonstrated (Guo et al. 2015).

#### 2.3.4 Resilience

Resilience was measured by one of the most popular resilience scales, the Brief Resilience Scale (BRS) (Chinese version: Fung (2020); Thai version: our translation based on the English version). The participants indicated how well the six statements described their behaviour and actions on a 5‐point Likert scale, ranging from 1 (Strongly disagree) to 5 (Strongly agree). Items included, ‘I tend to bounce back quickly after hard times.’ ‘It does not take me long to recover from a stressful event.’ and ‘I usually come through difficult times with little trouble.’ Items 2, 4 and 6 are reversely scored. The summation of all item scores yields the total resilience score, which ranges from 6 to 30. A higher score indicates better resilience. The Chinese version has been shown to have good criterion validity and internal consistency, with Cronbach's α of 0.71 in young adults (Fung 2020).

#### 2.3.5 Self‐Compassion

The study of self‐compassion was operationalised by the Chinese and Thai versions of the Self‐Compassion Scale (SCS) (Chinese version: Chen et al. (2011); Thai version: our translation based on the English version). The SCS is a 26‐item self‐administered questionnaire measuring six components of self‐compassion, containing five items each for self‐kindness and self‐judgement and four items each for common humanity, isolation, mindfulness and over‐identification. The items included, ‘I'm disappointing and judgemental about my own flaws and inadequacies’, ‘When things are going badly for me, I see the difficulties as part of life that everyone goes through’, ‘When times are really difficult, I tend to be tough on myself’ and ‘I'm intolerant and impatient towards those aspects of my personality I don't like’. The participants rated how often they felt or behaved in the manner stated in each item on a 5‐point Likert scale, where 1 stands for ‘Strongly disagree’ and 5 stands for ‘Strongly agree’. The items belonging to the three negative subscales were reversely scored. The six component subscale scores were calculated by summing the scores for the corresponding items. The total self‐compassion score, indicating the overall level of self‐compassion of the participant, was calculated by dividing the sum total of all item scores by the total number of items. The total self‐compassion scores ranged between 1 and 5. A higher total score indicates a higher level of self‐compassion. Good construct validity and internal consistency and test–retest reliability have been demonstrated in young adults (Chen et al. 2011).

To obtain a Thai version of the MHC‐SF, SCS and BRS, a back‐translation process was carried out by bilingual translators. Each scale was translated from English into Thai by two independent translators. The Thai versions were then backtranslated into English by two other independent translators who were unfamiliar with the concepts of the questionnaire. Face validity was conducted with 10 Thai university students to see whether Thai young adults understood the scale items. The translated scales were then revised and finalised based on feedback from the participants.

Tables 1 and 2 show the internal consistency of the scales used in the present study. Both the Thai and Chinese versions of the IGDS, MHC‐SF and SCS demonstrated good internal consistency, with Cronbach's α ranging from 0.72 to 0.95 and from 0.75 to 0.96, respectively. The internal consistency of both versions of the BRS was acceptable, with Cronbach's α of 0.61 in the Chinese version and 0.65 in the Thai version.

**TABLE 1:** Internal consistency of the Chinese version of the scales in the present study (*N* = 1450).

Scales	Variables	Item numbers on variable	Cronbach's alpha in the testing sample	Possible range
IGDS	Risk of Gaming Disorder	9	0.93	0–9
MHC‐SF	Positive mental health	14	0.95	0–70
Component 1	Emotion wellbeing	3	0.92	0–15
Component 2	Social wellbeing	5	0.87	0–25
Component 3	Psychological wellbeing	6	0.93	0–30
BRS	Resilience	6	0.61	6–30
SCS	Self‐compassion	26	0.82	1–5
Component 1	Self‐kindness	5	0.82	5–25
Component 2	Self‐judgement	5	0.74	5–25
Component 3	Common humanity	4	0.74	4–20
Component 4	Isolation	4	0.79	4–20
Component 5	Mindfulness	4	0.80	4–20
Component 6	Over‐identification	4	0.72	4–20

Abbreviations: BRS, Brief Resilience Scale; IGDS, Internet Gaming Disorder Scale; MHC‐SF, Mental Health Continuum Short Form; SCS, Self‐compassion Scale.

**TABLE 2:** Internal consistency of the Thai version of the scales in the present study (*N* = 300).

Scales	Variables	Item numbers on variable	Cronbach's alpha in the testing sample	Possible range
IGDS	Risk of Gaming Disorder	9	0.76	0–9
MHC‐SF	Positive mental health	14	0.96	0–70
Component 1	Emotion wellbeing	3	0.92	0–15
Component 2	Social wellbeing	5	0.87	0–25
Component 3	Psychological wellbeing	6	0.93	0–30
BRS	Resilience	6	0.65	6–30
SCS	Self‐compassion	26	0.75	1–5
Component 1	Self‐kindness	5	0.83	5–25
Component 2	Self‐judgement	5	0.68	5–25
Component 3	Common humanity	4	0.75	4–20
Component 4	Isolation	4	0.82	4–20
Component 5	Mindfulness	4	0.79	4–20
Component 6	Over‐identification	4	0.86	4–20

Abbreviations: BRS, Brief Resilience Scale; IGDS, Internet Gaming Disorder Scale; MHC‐SF, Mental Health Continuum Short Form; SCS, Self‐compassion Scale.

#### 2.3.6 Demographic Variables

Age, gender, living condition, occupation and education level were collected to obtain baseline characteristics of the participants. Age was collected as continuous data, education level was ordinal data and the others were categorical.

### 2.4 Statistical Analyses

Frequency and percentage were reported for ordinal and nominal variables such as gender, education level, gaming behaviour, positive mental health and level of GD risk. The median and interquartile ranges were computed for age, total resilience score, total and subscale scores of self‐compassion and total GD score because they were not normally distributed. The Kruskal–Wallis ANOVA with posthoc Mann–Whitney U test was used to compare differences between the three gamer groups (normal, at‐risk and disordered gamers) for ordinal variables, including education level, years of gaming, daily gaming time and positive mental health. It was also used to examine differences between the groups for age, self‐compassion scores and subscale scores and total resilience. The Chi‐squared test was used to examine intergroup differences regarding gender, living conditions and occupation. The Mann–Whitney U test was used to compare the differences of the three positive psychological variables between China and Thailand. To verify the hypotheses, the General Linear Model was used to compute the associations between the total GD score, total self‐compassion score, total resilience score and positive mental health. Gender and daily gaming time were included in the modelling because they showed significant differences between groups and they were consistently reported to have significant relationships with GD (Zhuang et al. 2023). Confidence intervals of 95% were reported. As there were no interaction effects between the two countries and the three positive psychological variables, subgroup analyses were not performed. SPSS version 26 was used to conduct all analyses. Statistical significance was set at *p* < 0.05.

### 2.5 Ethics

The study was carried out in accordance with the Declaration of Helsinki. It was approved by the Ethics Review Committee of Tung Wah College (REC2021106) and the corresponding institution or university in each region (Macau: RP/ESCSD‐03/2021; Lanzhou: LZUHLXY20210064; Shandong: 2021‐R‐043; Chiang Rai: E2565‐064). Consent to participate in the survey was collected online. The participants checked a box to signify their agreement with the statement ‘I read through the information above and agree to participate in the study’ prior to the commencement of the survey.

## 3 Results

A total of 1762 participants completed the questionnaire. The response rate could not be calculated because the project team did not have information about the number of people that received the survey. Out of the 1762 returned questionnaires, 12 were excluded due to invalid responses. Among the remaining 1750 participants with completed responses, the majority of them were from China (*n* = 1450, 82.9%) while the Thai participants were 17.1% (*n* = 300) of the sample. Males were less than half of the sample (*n* = 701, 40.1%) and the median age was 21 (IQR = 5). Over 70% of the participants were at the baccalaureate level and 51.8% of them were full‐time students.

### 3.1 Differences in Demographic and Gaming‐Related Variables Among the Three Gamer Groups

As illustrated in Table 3, the disordered gamer group had a significantly greater proportion of male participants (*n* = 218, 59.2%) while female participants were in the majority in both the normal and the at‐risk gamer groups. Around 61% of the disordered gamers were living with family compared with those below 20% and 40% in the normal and at‐risk gamer groups, respectively. A significantly greater proportion of disordered gamers were working in either full‐time or part‐time jobs compared with only around 15% and 8%, respectively, in the normal and at‐risk gamer groups.

**TABLE 3:** Comparison among the three risk levels of Gaming Disorder against baseline characteristics (*N* = 1750).

Demographic variables	Normal gamers (*n* = 890)	At‐risk gamers (*n* = 492)	Disordered gamers (*n* = 368)	Test statistics	*p* (a/b/c)
Country, *n* (%)					
China	743 (83.4)	363 (73.8)	344 (93.5)	χ^2^ = 58	0.00* (0.00/0.00/0.00)
Thailand	147 (16.5)	129 (26.2)	24 (6.5)		
Age (years), median (IQR)	21 (5)	21 (4)	22 (5)	H = 25.2	0.00* (0.00/0.02/0.00)
Gender, *n* (%)					
Male	263 (29.6)	220 (44.7)	218 (59.2)	χ^2^ = 255	0.00* (0.00/0.00/0.00)
Female	627 (70.4)	272 (55.3)	150 (40.8)		
Educational level,^a^ *n* (%)					
Secondary school or below	61 (6.8)	25 (5.1)	21 (5.7)	H = 73.8	0.00* (0.12/0.00/0.00)
Sub‐degree, e.g., Associate Degree	29 (3.3)	26 (5.3)	93 (25.3)		
Bachelor's degree	647 (72.7)	377 (76.6)	224 (60.9)		
Master's degree or above	152 (17.1)	63 (12.8)	30 (8.2)		
Living condition,^a^ *n* (%)					
Living alone	278 (31.2)	43 (8.7)	37 (10.1)	χ^2^ = 260.5	0.00* (0.00/0.00/0.00)
Living with family	158 (17.8)	176 (35.8)	224 (60.9)		
Living with friends	195 (21.9)	133 (27)	49 (13.3)		
Occupation, *n* (%)					
Full‐time student	473 (53.1)	284 (57.7)	150 (40.8)	χ^2^ = 243.3	0.00* (0.00/0.39/0.00)
Full‐time work	57 (6.4)	48 (9.8)	81 (22)		
Part‐time work	266 (2)	155 (5.3)	129 (20.9)		
Unemployed	94 (10.6)	5 (1)	8 (2.2)		
Gaming‐related variables					
Years of gaming,^a^ *n* (%)					
1–3 years	348 (53.1)	110 (22.4)	40 (10.9)	H = 223.4	0.00* (0.00/0.00/0.00)
4–6 years	86 (9.7)	73 (14.8)	64 (17.4)		
7–9 years	151 (17)	40 (8.1)	49 (13.3)		
≥ 10 years	56 (6.3)	139 (28.3)	163 (44.3)		
Daily gaming time over the past year^a^, *n* (%)					
1–2 h	586 (65.8)	275 (55.9)	88 (23.9)	H = 406.2	0.00* (0.00/0.00/0.00)
3–6 h	35 (3.9)	62 (12.6)	176 (47.8)		
7 h or above	20 (2.2)	25 (5.1)	52 (14.2)		

*Note:* a: *p* value between normal and at‐risk gamers. b: *p* value between normal and disordered gamers. c: *p* value between at‐risk and disordered gamers.

Abbreviations: χ^2^, Chi‐squared test; H, Kruskal Wallis ANOVA; IQR, Interquartile range.

^a^ Missing data.

* *p* < 0.001.

There were significant differences between the three groups as regards years of gaming and daily gaming time (Table 3). Post hoc analyses showed that disordered gamers had more years of gaming than normal and at‐risk gamers. Of the disordered gamers, 44.3% had played video and digital games for more than 10 years, while the participants who had played for more than 10 years in the at‐risk and normal groups were only 28% and 6%, respectively. The proportion of disordered gamers who had played for 7 years or more was 57.6% (*n* = 212), which was significantly greater than that in the normal gamer group (*n* = 207, 23.3%) and the at‐risk group (*n* = 179, 36.3%). Over 60% of the disordered gamers spent 3 h or more a day playing games, which was far more than the proportion in the other two groups.

### 3.2 Differences in Positive Mental Health, Self‐Compassion and Resilience Between the Three Gamer Groups and the Two Countries

All three positive psychological variables were found to have significant differences between the normal, at‐risk and disordered gamer groups. Post hoc analyses indicated that the total scores for self‐compassion and resilience among disordered gamers were significantly lower than among normal gamers (*p* < 0.00, 0.00 respectively). The subscale scores of self‐compassion among disordered gamers were also significantly lower than those among normal gamers, with the exceptions of common humanity and mindfulness. Among at‐risk gamers, only the isolation subscale score was found to be significantly lower than that of normal gamers (*p* < 0.04), while other subscale scores showed no significant differences between the two groups (see Table 4). Furthermore, the disordered gamers were found to have significant differences in positive mental health compared with normal and at‐risk gamers (*p* < 0.00, 0.00, respectively). Nearly 70% of the disordered gamers had moderate positive mental health as compared to 52.1% and 57.3% in normal and at‐risk gamers respectively. A significantly higher proportion of languishing mental health was observed in disordered gamers (17.9%) as compared to normal and at‐risk gamers (15.4%, 10%, respectively). The proportion of disordered gamers with flourishing mental health (13.3%) was lower than in the other two gamer groups.

**TABLE 4:** Comparison among the three risk levels of Gaming Disorder against independent variables (*N* = 1750).

Independent variables	Normal gamers (*n* = 890)	At‐risk gamers (*n* = 492)	Disordered gamers (*n* = 368)	H statistics	*p* (a/b/c)
Total GD score (ranged 0–9), Median (IQR)	0 (0)	3 (2)	9 (2)	1562.6	0.00** (0.00/0.00/0.00)
Positive mental health state, *n* (%)					
Flourishing	289 (32.4)	161 (32.7)	49 (13.3)	44.8	0.00** (0.18/0.00/0.00)
Moderate	464 (52.1)	282 (57.3)	253 (68.8)		
Languishing	137 (15.4)	49 (10.0)	66 (17.9)		
Self‐compassion, Median (IQR)					
Total score (ranged 1–5)	3.2 (0.5)	3.2 (0.5)	3 (0.4)	43.3	0.00** (0.08/0.00/0.00)
Self‐kindness subscale (ranged 5–25)	17 (4)	17 (4)	16 (4)	10.5	0.00* (0.66/0.00/0.00)
Common humanity subscale (range 4–20)	13 (4)	13 (4)	13 (4)	2.2	0.33
Mindfulness subscale (ranged 4–20)	14 (4)	14 (4)	14 (4)	2.4	0.31
Self‐judgement subscale (ranged 5–20)	15 (5)	15 (5)	14.5 (5)	28.8	0.00** (0.18/0.00/0.00)
Isolation subscale (ranged 4–20)	12 (5)	12 (5)	11 (4)	35.4	0.00** (0.04/0.00/0.00)
Over‐identification subscale (ranged 4–20)	12 (4)	12 (5)	11 (5)	21.6	0.00** (0.13/0.00/0.00)
Total resilience score (ranged 6–30)	19 (4)	19 (4)	18 (4)	14.3	0.00* (0.66/0.00/0.00)

*Note:* a: *p* value between normal and at‐risk gamers. b: *p* value between normal and disordered gamers. c: *p* value between at‐risk and disordered gamers.

Abbreviations: H, Kruskal Wallis ANOVA; IQR, Interquartile range.

* *p* < 0.01.

** *p* < 0.001.

Both positive mental health state and self‐compassion and its subscale scores were significantly different between the two countries (see Table 5). Approximately 40% of the Thai participants were in a flourishing state while only 14.8% were found among the Chinese participants (*p* < 0.00). The majority of the Chinese participants (i.e., 58.9%) were in the moderate mental health state. The total self‐compassion score in the Thai group was significantly greater than that of the Chinese group (U = 168,609, *p* < 0.00). Its six subscale scores were also significantly different between the two countries. The Thai participants were found to have significantly lower scores for the three positive subscales as compared with the Chinese participants (*p* < 0.00). Opposite results were observed in the three negative subscales (*p* < 0.00).

**TABLE 5:** Comparison of the three positive psychological variables between Thailand and China (*N* = 1750).

Independent variables	China (*n* = 1450)	Thailand (*n* = 300)	U statistics	*p*
Positive mental health state, *n* (%)				
Flourishing	381 (14.8)	118 (39.3)		0.00**
Moderate	854 (58.9)	145 (48.3)	189,311.5	
Languishing	215 (14.8)	37 (12.3)		
Self‐compassion, median (IQR)				
Total score (ranged 1–5)	3.1 (0.5)	3.2 (0.6)	168,609	0.00**
Self‐kindness subscale (ranged 5–25)	17 (4)	16 (6)	193,895	0.00*
Common humanity subscale (range 4–20)	13 (4)	12 (5)	158,386	0.00**
Mindfulness subscale (ranged 4–20)	14 (4)	13 (6)	186,201	0.00**
Self‐judgement subscale (ranged 5–20)	15 (5)	18 (5)	116,701.5	0.00**
Isolation subscale (ranged 4–20)	12 (5)	15 (5)	117,555.5	0.00**
Over‐identification subscale (ranged 4–20)	12 (5)	15 (4)	130,606	0.00**
Total resilience score (ranged 6–30)	19 (4)	19 (3)	213,722.5	0.63

Abbreviations: IQR, Interquartile range; U, Mann–Whitney U test.

* *p* < 0.01.

** *p* < 0.001.

### 3.3 Associations Between Risk of GD, Self‐Compassion, Resilience and Positive Mental Health

Table 6 presents the regression model for the association of the risk of GD with resilience, self‐compassion and positive mental health with an *R* ^2^ of 0.35 (*F* = 102.80, *p* < 0.001). It is shown that 35% of the variance of the model can be explained by the five independent variables. The effect size (*f* ^2^) of the constructed model was 0.54, which represents a large effect (Cohen 1992). Male gamers are at significantly greater risk of GD as compared to female gamers (B [95% CI] = 0.74 [0.44, 1.05]). Gamers who played games for 1–2 h a day had significantly less risk of GD than those who played games for ≥ 7 h a day (B [95% CI] = −3.20 [−3.78, −2.62]). However, gamers who spent 3–6 h gaming each day were at significantly greater risk of GD than those with daily gaming time ≥ 7 h (B [95% CI] = 0.81 [0.19, 1.44]). As regards the three positive psychological variables, young adult gamers with moderate mental health had a significantly greater risk of GD than those with flourishing mental health (B [95% CI] = 0.52 [0.18, 0.86]). The total self‐compassion score was negatively associated with the risk of GD (B [95% CI] = −0.48 [−0.91, −0.06]). The total resilience score was not significantly associated with the risk of GD (*p* = 0.06). The results indicate that young adult gamers having moderate mental health and higher levels of self‐compassion exhibit a lower risk of GD.

**TABLE 6:** The results of the General Linear Model for the associations between the total GD score, gender, daily gaming time and the three positive psychological variables (*N* = 1319).

Independent variables	B	SE	95% CI	*p*
Gender				
Male	0.74	0.16	0.44, 1.05	0.00***
Female	Ref.			
Daily gaming time over the past year				
1–2 h	−3.20	0.29	−3.78, −2.62	0.00***
3–6 h	0.81	0.32	0.19, 1.44	0.01*
7–10 h	Ref.			
Positive mental health state				
Languishing	0.04	0.25	−0.45, 0.54	0.86
Moderate mental health	0.52	0.18	0.18, 0.86	0.00**
Flourishing	Ref.			
Total resilience score	−0.05	0.03	−0.1, 0.002	0.06
Total self‐compassion score	−0.48	0.22	−0.91, −0.06	0.03*
*F*				102.80
*p* value				0.00***
Adjusted *R* ^2^				0.35
*f* ^2^				0.54

Abbreviation: 95% CI, 95% confidence interval.

* *p* < 0.05.

** *p* < 0.01.

*** *p* < 0.001.

## 4 Discussion

The significant negative association between self‐compassion, positive mental health and risk of GD supported Hypothesis 1 and Hypothesis 3 while the insignificant association between resilience and risk of GD rejected Hypothesis 2. The constructed regression model suggests that young adult gamers with low self‐compassion and moderate mental health may be at greater risk of addictive gaming. These findings are of significant research and practical value as the constructed model is the first to reveal the negative connections between self‐compassion, positive mental health and GD. The significant negative associations imply the potential protective effects of positive mental health against addictive gaming, which have not been reported in previous studies. This study points the way for mental health researchers to study GD using a positive psychological approach in addition to the predominant psychopathological approach. Frontline primary healthcare stakeholders, including mental health nurses, social workers and psychologists, could screen for at‐risk individuals based on their level of self‐compassion and positive mental health in the community setting in order to achieve early identification and intervention.

The negative association between GD and self‐compassion might be explained by the importance of self‐compassion for emotion regulation. Existing evidence generally posited that people with lower ability to regulate emotion tend to suffer from addictive gaming because, firstly, they will tend to experience greater stress and negative emotion when facing adversities in life and, secondly, the lower ability to regulate emotion may bias their affective and cognitive responses to gaming‐related cues and so may lead to uncontrollable or even addictive gaming to cope with their stress and negative emotion (Brand et al. 2016). Enhancing the ability to regulate emotion may therefore be an effective approach for reducing the risk of GD. Self‐compassion has recently been highlighted as a positive coping strategy to manage stress, negative emotion and depression (Diedrich et al. 2014; Neff and Dahm 2015). Evidence has generally supported the idea that the positive components of self‐compassion contribute to emotion regulation because individuals who are self‐compassionate treat themselves with care and kindness (self‐kindness), perceive negative experiences in daily life as a shared aspect of human experience (common humanity) and tend to remain mindful and accepting of whatever they are experiencing at the present moment (mindfulness). These abilities help to develop positive coping styles (Neff et al. 2007; Neff and Dahm 2015). However, the idea is not fully supported by the findings of the present study which showed that only one component of self‐compassion, namely self‐kindness, differed significantly between the normal and disordered gamer groups. Similarly, Iskender and Akin (2011) reported that only self‐kindness and mindfulness were negatively associated with Internet addiction in university students. Regarding the negative subscales (i.e., over‐identification, self‐judgement, isolation), the present study found significant differences between the groups, which are congruent with those reported in the study of Internet addiction (Iskender and Akin 2011). The difference in the contributions of positive and negative subscales to GD adds to the existing knowledge that not all positive components of self‐compassion contribute strongly to GD. More studies are needed to verify the contribution of each positive and negative component of self‐compassion to GD.

The significant negative association between positive mental health and risk of GD suggests that GD is not impacted solely by psychopathological outcomes, which are well‐established psychological factors contributing to Internet‐related disorders (Brand et al. 2016), but also by positive mental health. The finding implies that people with moderate mental health could be at risk of GD as compared to those with flourishing mental health. This is congruent with previous studies of Internet‐related addictive problems (Brailovskaia et al. 2019; Brailovskaia and Margraf 2022). Growing evidence suggests that positive mental health moderated the impact of depression on suicide ideation. Teismann et al. (2018) found that the level of depression in young adults with a higher level of positive mental health had no association with suicide ideation over time. Some other studies reported that higher levels of positive mental health can facilitate remission of mental disorders (Lukat et al. 2017) and were negatively associated with adjustment disorder symptoms (Truskauskaite‐Kuneviciene et al. 2022). This negative association may be related to stress and emotion management among gamers. People with flourishing mental health can regulate their emotion in an effective way when facing stressful events (Morrish et al. 2018). The ability to up‐regulate positive emotion has been found to be as important for maintaining and improving mental well‐being as the ability to down‐regulate negative emotion (Gruber et al. 2013; Quoidbach et al. 2015). People with poor emotion regulation often engage in maladaptive behaviours to escape their negative emotion with the consequent risk of additive disorders (Williams et al. 2012). Young adult gamers who are more able to manage stress and increase positive emotion through effective coping strategies could reduce their risk of depression, anxiety and hostility (Yen et al. 2017) and eliminate GD risk factors. Future studies shall include constructs related to emotion regulation, such as positive emotion and coping style.

Previous studies found that resilience was negatively associated with Internet Gaming Disorder (Shin et al. 2019; Yen et al. 2019) and other Internet‐related addictions (e.g., Nam et al. 2018; Žmavc et al. 2022), which contradicts the present finding. Our study showed that resilience was not significantly associated with GD. There are two possible reasons for the discrepant findings. Firstly, a recent systematic review and meta‐analysis reported that resilience had a weak correlation (*r* = −0.27) with Internet‐related addiction (Hidalgo‐Fuentes et al. 2023). This implies that there might be other positive psychological factors that strongly influence the addiction risk. Resilience is perceived as an important factor in psychological well‐being and is positively correlated with positive psychological factors such as life satisfaction and positive affect (Hu et al. 2015). However, the previous studies seldom included other positive psychological factors when examining the association between resilience and Internet‐related disorders. In the present study, self‐compassion and positive mental health are two other positive psychological factors that are related to psychological well‐being, in addition to resilience. The moderate correlation between self‐compassion and resilience found in the present study may overlay the effect of resilience on the risk of GD. Secondly, the incongruent finding may be caused by the different measurement tools used in the present and previous studies. The present study used the Brief Resilience Scale (BRS) developed by Smith et al. (2008). The BRS operationalises resilience as an individual's ability to bounce back from adverse situations without considering the availability of psychological resources and external resources, which are measured in other resource‐based resilience scales, for example, the Connor‐Davidson Resilience Scale and the Resilience Scale (Ye et al. 2022). As the operationalizations of resilience using BRS and other resource‐based resilience scales are inherently different, their use may yield different results and different implications. Previous studies used the resource‐based resilience scales to measure resilience and their significant findings have indicated that the availability and adequacy of internal and external resources to manage stress and adversity are crucial for mitigating GD (Shin et al. 2019; Yen et al. 2019), although those studies suffered from some methodological issues, as explained earlier. In the present study, the participants' responses to BRS only reflect the participants' self‐efficacy to manage stress. The very small unstandardised coefficient (B) (B = –0.07, *p* = 0.002) when the total resilience score was input alone to the regression model implies that a person's perceived ability to manage stress has little influence on GD.

The present study has three major limitations. First, it was a cross‐sectional study and could not establish a causal relationship since the design did not allow for investigation of the temporality of the study variables. A longitudinal design should be used in future studies to verify the causal relationship between the three positive psychological factors and the risk of GD. Nonetheless, the present study provides preliminary evidence to support such an association, laying a foundation for future research and indicating to healthcare professionals that screening and preventive intervention for GD can usefully incorporate a positive psychological approach in addition to the psychopathological approach. The second limitation is that the present study only recruited participants from Thailand and China. The findings may not extend to other countries as only self‐compassion has been found to be a stable construct across different cultures (Neff et al. 2008), but not resilience (Ungar 2015) or positive mental health (Vaillant 2012). A larger study involving samples from different countries or ethnic groups is needed in order to verify the present findings. Thirdly, the self‐reported approach often suffers from response biases, such as the social desirability bias. In this regard, the survey used a self‐administered approach and was made anonymous in order to minimise potential biases. Future studies should use more than one measurement tool to measure the same variable in order to triangulate the findings.

## 5 Conclusions

The present study supports the associations between self‐compassion, positive mental health and risk of GD, but not between resilience and risk of GD. The findings indicate that self‐compassion and positive mental health are negatively associated with the risk of GD while resilience has no association with the risk of GD. Such findings suggest, at the least, that resilience may not be the most important associated factor with GD when other positive psychological factors are considered together with it. While further studies are needed to verify such findings by including more diverse samples and using a longitudinal approach to explore the causal relationship between the variables, the present study supplements the existing understanding of the influence of positive psychological factors on Internet‐related disorders including GD. Furthermore, the findings could encourage primary healthcare professionals to pay attention to levels of self‐compassion and positive mental health among young adult gamers when conducting health screening, since this could enable earlier identification of individuals who are at risk of addictive gaming.

## Author Contributions

Anson Chui Yan Tang: Conceptualization, Funding acquisition, Methodology, Project administration, Formal analysis, writing – original draft and editing. Regina Lai‐Tong Lee: Conceptualization, Methodology, data curation, writing – original draft and editing. Hong Lee: Methodology, formal analysis, writing – original draft and editing. Winnie Lai Sheung Cheng: Investigation, Methodology, data curation, writing – review and editing. Chan Shun: data curation, formal analysis, writing – review and editing. Yufang Guo: Investigation, project administration, data curation, writing – review and editing. Yan Wang: Investigation, project administration, data curation, writing – review and editing. Qing Wang: Investigation, project administration, data curation, writing – review and editing. Wongchaiya Pimpimon: Investigation, Project administration, data curation, writing – review and editing. Lorna Kwai Ping Suen: Conceptualization, Funding acquisition, Methodology, Formal analysis.

## Funding

This work was supported by Tung Wah College (2021‐04‐52‐SRG210402).

## Ethics Statement

The study was carried out in accordance with the Declaration of Helsinki. It was approved by the Ethics Review Committee of Tung Wah College (REC2021106) and the corresponding institution or university in each region (Macau: RP/ESCSD‐03/2021; Lanzhou: LZUHLXY20210064; Shandong: 2021‐R‐043; Chiang Rai: E2565‐064).

## Consent

Informed consent was obtained from the participants online before the online survey started.

## Conflicts of Interest

The authors declare no conflicts of interest. There is a statistician on the author team. He is Dr. Paul Hong Lee, from the University of Southampton, the United Kingdom.

## Data Availability

The data that support the findings of this study are available from the corresponding author upon reasonable request.
